# Design and Optimization of a New Alternating Electromagnetic-Field-Generation System for an Inverted Microscope

**DOI:** 10.3390/mi13040542

**Published:** 2022-03-30

**Authors:** Zehao Wu, Ziheng Xu, Qingsong Xu

**Affiliations:** 1 Department of Electromechanical Engineering, Faculty of Science and Technology, University of Macau, Avenida da Universidade, Taipa, Macau, China; yb97489@um.edu.mo; 2 Pui Ching Middle School, Avenida de Horta e Costa, No. 7, Macau, China; s11012757@imail.puiching.edu.mo

**Keywords:** microrobotics, rotational electromagnetic system, alternating magnetic field, rolling microparticle

## Abstract

This paper presents the design and optimization of a new alternating electromagnetic-field-generation system, which is dedicated to actuating untethered magnetic microrobots under an inverted microscope. Its uniqueness is that the system parameters are optimally designed by considering both electric and geometry constraints for the target-driving application. The dominant parameters of the system are first determined by establishing analytical models. According to the requirements of targeted application, the optimization problem with certain constraints is formulated, which is solved via the multiobjective genetic algorithm method. A prototype system with the optimal parameters is developed for experimental testing. Experimental studies are carried out to characterize actual performance of the developed actuation system. For demonstration, a magnetic microball has been actuated for navigation by surface rolling in a petri dish filled with pure water. Results indicate that the reported electromagnetic-field-generation system meets the actuation requirements for potential applications.

## 1. Introduction

Over the past decade, precision medicine has drawn great attention from researchers. For the problem of precisely delivering medicine to hard-to-reach regions in human body, untethered microrobots provide a promising solution. The untethered microrobots are expected to reach the organs and tissues inside human body through the circulatory system [1,2]. Generally, there are four main types of untethered microrobots driven by optical tweezers [3], electrostatic fields [4], acoustic fields [5], and magnetic fields [6], respectively. In particular, magnetic microrobots have a great advantage in wireless driving, penetration into biological entities, and biocompability [7,8]. In the literature, a number of magnetic microrobots have been reported for biomedicine, e.g., drug delivery [9,10], gastrointestinal inspection [11], and cell manipulation [12,13].

In general, there are two common types of driving methods for magnetic microrobots, including alternating magnetic flux actuation and gradient magnetic flux density actuation. For the gradient magnetic flux density actuation, a magnetic field with a potential energy difference is generated, by which the magnetic microrobot can move from a position with higher potential energy to other position with lower potential energy. This method makes no specific requirements regarding the shape of the microrobots. However, it is difficult to generate a magnetic field with a large potential energy gradient to impose a sufficient actuation force on the microrobot [14,15]. For the alternating magnetic flux actuation, an alternating magnetic flux is exerted on the magnetic microrobot, and the microrobot is driven to navigate by rolling motion, helical propulsion, or swing propulsion, which is induced by the exerted magnetic torque. Moreover, in the uniform area of the generated magnetic field, the alternating magnetic flux actuation can drive multiple (or swarm) microrobots synchronously, which can improve the efficiency of micromanipulation [16,17,18]. Herein, this work is focused on the alternating magnetic flux actuation system.

The performance of a magnetic actuation system is heavily dependent on mechanism design of the system, which governs the maximum magnetic flux density, efficient operation frequency range, and continual working time [19,20]. In the literature, a number of magnetic actuation systems have been designed by optimally tuning the parameters of electromagnetic coils. For instance, Ongaro et al. used parametric scanning approach to find the influence of coil parameters on the magnetic flux density [21]. To improve the magnetic flux density and gradient, Li et al. adopted ANSYS software to optimally adjust the geometry of electromagnet cores [22]. In the existing designs, only the geometry parameters of the coils (including radius and height) have been considered. Actually, the system’s actuation performance is also influenced by the electric parameters, such as inductance and maximum working current. The inductance has a direct influence on the cut-off frequency (in open loop). Thus, a large inductance indicates that the system requires a large phase-lead compensation when the microrobot needs an actuation magnetic field with high frequency [23]. Although it is easy to improve the magnetic flux intensity by increasing the input current, the heating power generated by applying an exorbitant input current is prone to burn out the system [24,25]. The heating power will also enlarge the resistivity of the coils, and increases the power-supply burden of the system accordingly.

Moreover, there are two popular types of cores adopted in electromagnetic coils, i.e., air core and metal core. The air core provides much uniform field, whereas its magnetic flux density is relatively low [26,27]. The metal core can offer a relatively large magnetic flux density, however, the unwanted field gradient and inductance are also increased [21,28]. In the literature, the work on design optimization of alternating electromagnetic field generation systems is fewer than that on gradient electromagnetic field generation systems. Recently, by considering both electric and geometry issues, an alternating electromagnetic field generation system was optimally designed to generate a magnetic field with high magnetic flux density and relatively uniform gradient [29]. However, there was a relatively plarge discrepancy between simulation and experimental results, due to the poor estimation of geometry parameters based on the number of turns for the coils [29]. The inductances of coils and the direction change of magnetic flux in the workspace were not considered in the optimization. For practical use, it is desirable to realize more accurate parameter estimation and a sophisticated optimization process to determine the optimal parameters for an alternating electromagnetic-field-generation system.

Herein, the main contribution of the paper is the optimal design of an alternating electromagnetic-field-generation system in consideration of both electrical and geometric aspects. The system is targeted at driving magnetic microrobots under an inverted microscope for biological applications. In view of the requirements of the targeted application under an inverted microscope, optimization objectives and constraints are assigned, and design optimization is conducted by finite-element analysis software with an embedded genetic algorithm for determining the optimum parameters of the alternating electromagnetic-field-generation system. Based on the optimal parameters, a prototype of the proposed system has been fabricated. Experimental tests were conducted to verify the real performance of the developed system.

The remaining parts of the paper are organized as follows. The design and modeling of the alternating electromagnetic field generation system are outlined in Section 2. The optimization setup and optimization result are presented in Section 3. Section 4 reports the prototype fabrication and experimental studies. Section 5 concludes the paper.

## 2. Mechanism Design and Analytical Modeling

In this section, the mechanism design of the proposed alternating magnetic-field-generation system is presented. Analytical models are derived for assessing the system performance.

### 2.1. Mechanism Design of the Alternating Electromagnetic Field Generation System

**Figure 1:**
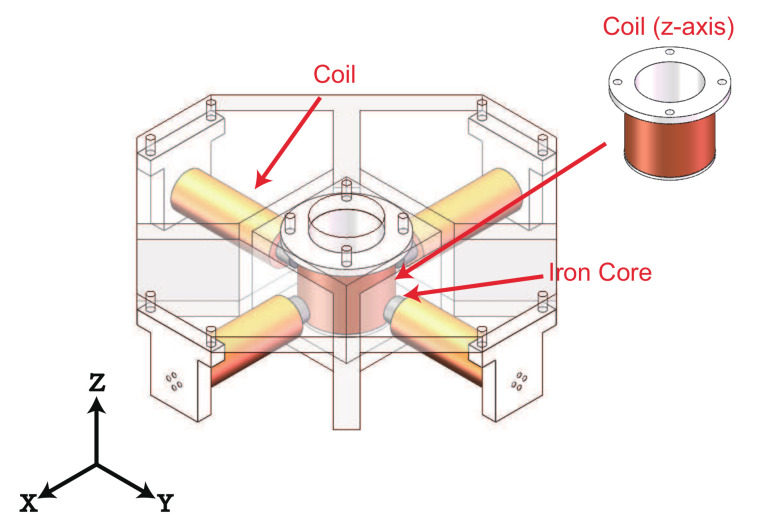
CAD model of the designed alternating magnetic field generation system with five electromagnetic coils.

As shown in Figure 1, two couples of electromagnetic coils with iron cores are adopted to generate the components of magnetic field along the x-axis and y-axis, separately. In addition, an electromagnetic coil with air core is used to generate the component of magnetic field along the z-axis. To yield the magnetic field in any direction in 3-D space, the electromagnetic coils of each axis in this work are orthogonal to the corresponding electromagnetic coils of other axes. The magnitudes and the direction of the magnetic field generated by the coils can be adjusted by changing the magnitude and the direction of the input currents for the coils, respectively. In this way, the desired magnetic field can be easily generated. The workspace is set at the bottom of air core of the coil along z-axis. The size of the expected workspace is determined as 10 × 10 × 10 mm. As the electromagnetic coils along the x-axis and y-axis are away from the workspace (with certain distance), the iron cores are inserted to improve the densities of the generated magnetic fields. Meanwhile, the iron cores increase the inductances of the electromagnetic coils along the x-axis and y-axis.

### 2.2. Modeling of Magnetic Field and Rotating Object

The magnetic flux density (B) is determined based on the Maxwell’s equations, which are expressed below.

(1) $$\begin{array}{c} \left\{\begin{array}{c} \nabla \;\cdot \;B=0\\ \nabla \times B=\mu J \end{array}\right.\; \end{array}$$

where μ is the magnetic permeability and **J** is the current density.

**Figure 2:**
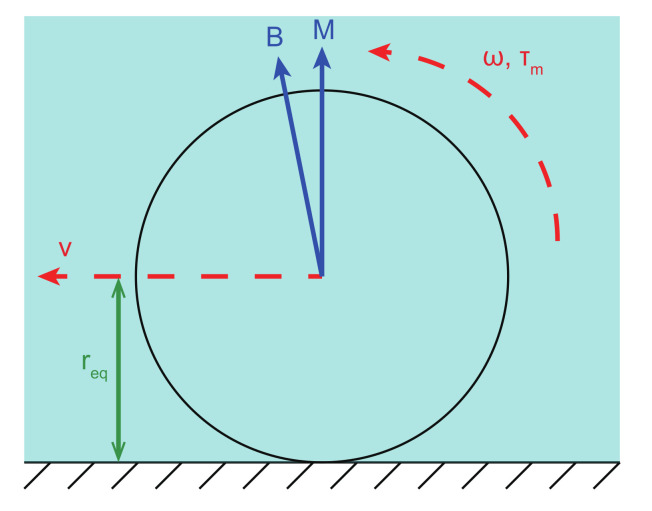
Free-body diagram of a rolling microparticle.

As depicted in Figure 2, a microparticle with an overall size larger than 10 μm (functions as a microrobot) is actuated to roll forward by a magnetic torque generated from an alternating magnetic actuation system. In addition, if the overall size of the microparticle is less than 10 μm, the motion of the rolling microparticle is always slipping motion, since there will be a lubrication layer between the microparticle and the wall [30,31,32]. When the microparticle rotates in a flow environment with an angular velocity ω, its motion can be expressed by:

(2) $$v=\omega \;r_{eq}$$

(3) $$\tau_{r}=\tau_{d}+\tau_{f}\;$$

where *v* and $r_{eq}$ denote the translation velocity relative to the ground and the equivalent radius of the microparticle, respectively. $\tau_{r}$ is the required actuation torque of the microparticle. $\tau_{f}$ denotes the spinning friction-based resistive torque of the microparticle. $\tau_{d}$ represents the viscosity drag torque, which is governed by the shape, angular velocity, and environment of the microparticle.

If the magnetic torque exerted on the microparticle ($\tau_{m}$) is smaller than $\tau_{r}$, then the microparticle will rotate in the step-out state, i.e., the rotation motion of the microparticle cannot follow the actuation frequency. Thus, $\tau_{m}$ can be determined as:

(4) $$\tau_{m}=M\times B$$

where M represents the magnetic moment of the microparticle. In addition, M and B are perpendicular to the rotating axis of the microparticle.

### 2.3. Modeling of Geometric and Electric Performance

**Figure 3:**
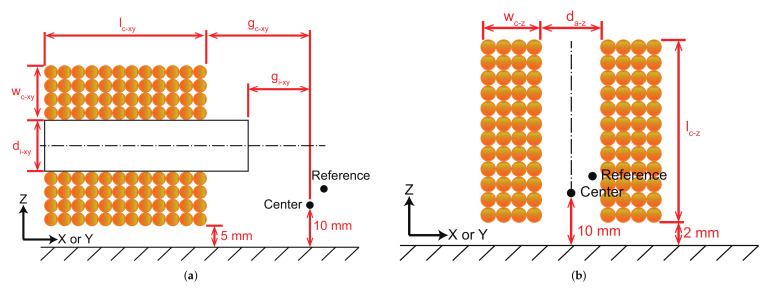
Main geometric parameters of the coils (**a**) along x-axis or y-axis, and (**b**) along z-axis.

The subscript dir represents the corresponding axis of the coils, which can be replaced by xy or *z*. The subscript xy means x-axis or y-axis, and the subscript *z* denotes z-axis. With reference to the coil arrangement as given in Figure 3, the parameters $l_{c-dir}$ and $w_{c-dir}$ can be expressed as follows.

(5) $$l_{c-dir}=N_{lc-dir}\left(d_{c-dir}+0.02\;\mathrm{mm}\right)$$

(6) $$w_{c-dir}=N_{wc-dir}\left(d_{c-dir}+0.02\;\mathrm{mm}\right)$$

where $N_{lc-dir}$ and $N_{wc-dir}$ denote the number of radial and axial turns, respectively. $d_{c-dir}$ describes the diameter of the copper wire without paint. In general, the paint will increase the diameter of the copper wire by 0.005–0.02 mm. In this work, the diameter increased by the paint is selected as 0.02 mm.

The resistance ($R_{dir}$) of the coils can be calculated by:

(7) $$R_{dir}=\alpha \rho \frac{l_{dir}}{S_{dir}}=\alpha \rho \frac{4N_{lc-dir}N_{wc-dir}\left(2w_{c-dir}+d_{dir}\right)}{d_{c-dir}^{2}}$$

where α denotes the temperature coefficient of resistance, which is related to the temperature of the working coils and the room environment. ρ is the resistivity of copper material. For the wire of the coils, the total length is represented as $l_{dir}$, and the effective cross-sectional area is recorded by $S_{dir}$. $d_{dir}$ is the diameter of the core ($d_{i-xy}$ and $d_{a-z}$).

From prior experience, with a sinusoidal driving signal, the maximum input current ($I_{c-dir}$) of the copper wire (which can be available for use) is determined as:

(8) $$I_{c-dir}=\sqrt{2}\left(10\;A/\mathrm{mm}{}^{2}\right)S_{dir}=\frac{\sqrt{2}}{4}\left(10\;A/\mathrm{mm}{}^{2}\right)\pi d_{c-dir}^{2}$$

Considering the current limit of servo amplifiers for the coils, the maximum working current ($I_{max-dir}$) of the coils is determined by:

(9) $$I_{max-dir}=\min\left(I_{c-dir},V_{b}/R_{dir-0},I_{b}\right)$$

where $V_{b}$ and $I_{b}$ are the maximum output voltage and maximum output current of the servo amplifiers, respectively. $R_{dir}$ is equal to $R_{dir-0}$ when α = 1.

In addition to the number of turns for the coils and the material of the core, the inductance ($L_{dir}$) and magnetic field generation ability ($B_{dir}$) are also correlated with the position and shape of the coils and core. Let *I* denote the input current of the coils. $B_{o-dir}$ is the magnetic flux density generated at the center point ([0, 0, 0]) with 1 A input current. Referring to Figure 3, some basic parameters are determined. Thus, $L_{dir}$ and $B_{o-dir}$ are both related to position and shape of the cores and the winding method of the coils, which can be expressed as follows.

(10) $$\begin{array}{cc} & L_{xy}\left(N_{lc-xy},N_{wc-xy},d_{i-xy},g_{c-xy},g_{i-xy},d_{c-xy}\right) \end{array}$$

(11) $$\begin{array}{cc} & B_{o-xy}\left(N_{lc-xy},N_{wc-xy},d_{i-xy},g_{c-xy},g_{i-xy},d_{c-xy}\right) \end{array}$$

(12) $$\begin{array}{cc} & L_{z}\left(N_{lc-z},N_{wc-z},d_{a-z},d_{c-z}\right) \end{array}$$

(13) $$\begin{array}{cc} & B_{o-z}\left(N_{lc-z},N_{wc-z},d_{a-z},d_{c-z}\right) \end{array}$$

where $g_{c-xy}$ and $g_{i-xy}$ are denoted in Figure 3a. Then, the maximum magnetic flux density generated at the center point ($B_{max-dir}$) can be calculated by:

(14) $$B_{max-dir}=B_{o-dir}I_{max-dir}/(1\;A)\;$$

The uniformity depends on the direction change and magnitude change of the magnetic field. To assess the uniformity of the magnetic field, a corner point ([5 mm, 5 mm, 5 mm]) in the expected workspace is selected as the reference point. Then, the uniformity can be determined as the magnitude of direction change ($A_{ang-dir}$) and magnitude change ($A_{grad-dir}$), which are expressed as follows.

(15) $$\begin{array}{ccc} A_{ang-dir} & = & \frac{B_{ref-dir}}{B_{ref}} \end{array}$$

(16) $$\begin{array}{ccc} A_{grad-dir} & = & \frac{|B_{o-dir}-B_{ref}|}{5\sqrt{3}\;\mathrm{mm}}\;\cdot \;\frac{I_{max-dir}}{1\;A} \end{array}$$

where $B_{ref-dir}$ is the magnetic flux density generated along the corresponding axis of the coils at the reference point with an input current of 1 A. $B_{ref}$ is the total magnetic flux density generated at the reference point with an input current of 1 A.

## 3. System Parameter Optimization

In this section, for determining optimal values of the system parameters, the optimization based on multiobjective genetic algorithm (MOGA) method is carried out with finite element analysis simulation study. In addition, the Pareto efficiency has been used to find the optimal equilibrium state. After optimization, manual reviewing and screening has been carried out to improve the result.

### 3.1. Optimization Setup

In this work, the performance of the coils was simulated by ANSYS software with magnetostatic module. Assuming the room temperature, two simulation studies were conducted for the coils along x-axis (or y-axis) and the coil along z-axis, respectively. According to the adopted servo amplifiers of the coils, the parameters $V_{b}$ and $I_{b}$ were assigned as 57 V (i.e., 95% of supply voltage) and 10 A, respectively. The main input parameters are denoted in Equations (10)–(). For illustration, the input current was set as 1 A in the simulation study.

In view of the requirements of targeted application, the optimization objectives and constraints are summarized in Table 1. Under a more uniform magnetic field, $A_{ang-dir}$ becomes closer to 1.0 and $A_{grad-dir}$ becomes smaller. Moreover, as compared with the size of coil along the z-axis, the size of workspace is relatively large and the gradient of the coil along the z-axis cannot be ignored. This issue is described by parameter $A_{ang-z}$. In particular, $A_{ang-dir}\geq$ 0.99 indicates that the inclined angle between the direction of magnetic field at the center point and reference point is less than $8.1^{{}^{\circ}}$. In addition, a larger $B_{max-dir}$ indicates a larger magnetic torque exerted on the microrobot. A smaller inductance provides a larger cut-off frequency (in open loop). Besides, the levels of importance in the embedded optimization module were set according to the desired application. The set of input parameter ranges is denoted as $R^{m}$.

### 3.2. Optimization Result

**Figure 4:**
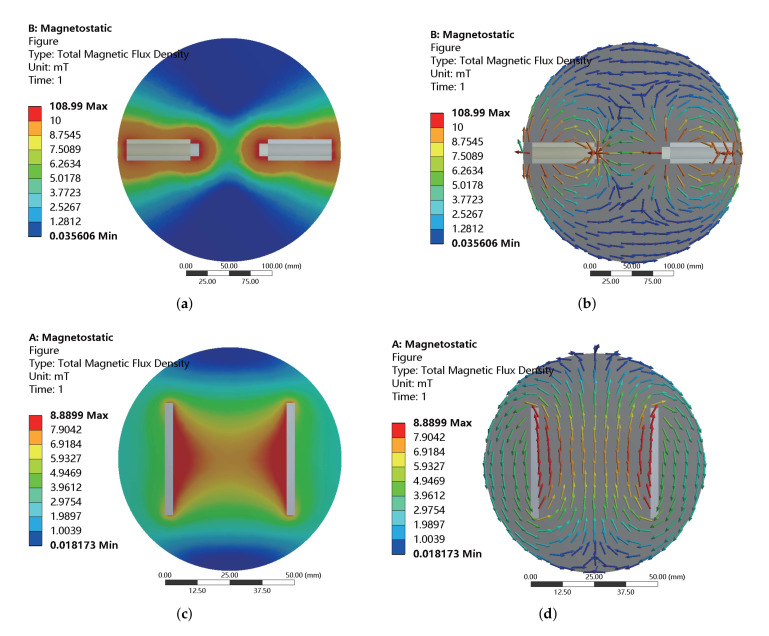
Simulation results of magnetic flux density distribution for the coils along (**a**,**b**) x-axis or y-axis, and (**c**,**d**) z-axis with an input current of 1 A. (**b**,**d**) Simulation results with vectorized representation.

Here, the embedded MOGA method of ANSYS software is adopted to optimally tune the system parameters. In addition, the accuracy of the optimization results is further improved by manual inspection and screening. For the foregoing optimization objectives and constraints, the optimization results are obtained as tabulated in Table 2. The maximum working currents of the coils along all axes are 4 A, which are the maximum input current of the copper wire with a diameter (without paint) of 0.6 mm. For the coils along the x-axis (or y-axis) and z-axis, the maximum magnetic flux densities generated at the center point are obtained as 20.8 mT and 23.6 mT, respectively. The magnitude changes are derived as 0.2 T/m and 0.5 T/m, respectively. The resistances are derived as 3.6 Ω and 3.1 Ω, respectively. The inclined angles between the direction of magnetic field at the center point and reference point for the coils along all axes are both less than $4.4^{{}^{\circ}}$. In addition, according to the influence of iron cores, the inductances of the coils along the x-axis (or y-axis) are much larger than the inductance of the coil along the z-axis. Under an input current of 1 A, the magnetic flux density distribution is obtained as given in Figure 4. It illustrates the magnetic flux density distribution for the coils along three axes. In the expected workspace, the magnetic flux densities are uniform and the directions of magnetic flux densities are aligned with their corresponding axes.

## 4. Prototype Fabrication and Performance Test

Based on the determined optimal parameters of the magnetic actuation system, a prototype has been fabricated and experimental tests have been conducted to evaluate the real performance of the developed alternating electromagnetic-field-generation system.

### 4.1. Prototype Fabrication and Experimental Setup

**Figure 5:**
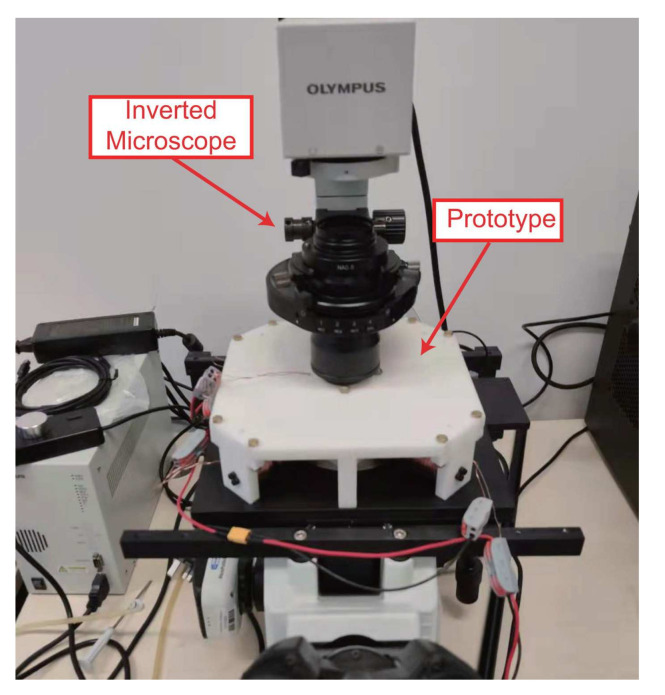
Prototype of the fabricated alternating magnetic actuation system installed on an inverted microscope.

Figure 5 gives a photo of the fabricated prototype, which is installed on an inverted microscope (model: IX-81, from Olympus Corp., Tokyo, Japan). The heat-resistance capability for the adopted copper wire of coils is 155 ${}^{{}^{\circ}}C$. The input current of the coils along three axes is supplied by three servo amplifiers (model: ESCON 70/10, from Maxon motor AG, Sachseln, Switzerland). A power supply (model: KXN-6020D, from Shenzhen Zhaoxin Electronic Instrument and Equipment Co., Ltd., Shenzhen, China) is adopted to power the servo amplifiers. The servo amplifiers are controlled by a personal computer (model: OptiPlex 9020, from Dell Technologies Inc., Round Rock, TX, USA) and a driving board (model: USB-6259, from National Instruments Corp., Austin, TX, USA), which offers analog input and analog output channels.

In the following experimental tests, the temperature was measured by an infrared thermometer, and the magnetic flux density was detected by a 3D magnetic sensor (model: TLV493D-A1B6, Infineon Technologies AG, Neubiberg, Germany). The inverted microscope is equipped with a CCD camera (model: micropublisher 5.0 RTV, Teledyne Photometrics Corp., Surrey, Canada). In addition, the resistances of the coils along x-axis and y-axis were both measured as 3.9 Ω, which is 8% larger than the simulation result. The resistance of the coil along the z-axis was measured as 3.2 Ω, which is 3% larger than the simulation result. Thus, the discrepancies between the actual and simulation results are relatively low.

### 4.2. Performance Test of the Continuing Working Time

**Figure 6:**
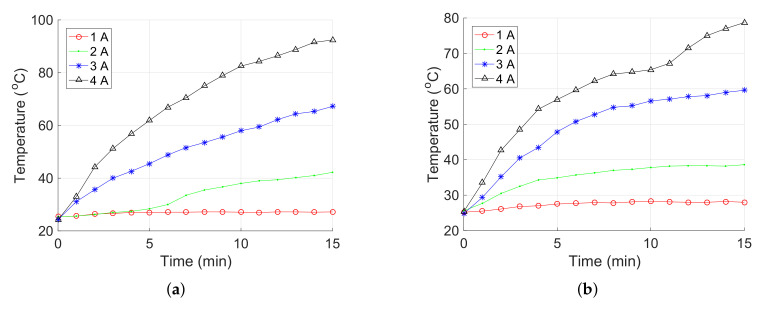
Relationship between the temperature for the electromagnetic coils along (**a**) x-axis or y-axis, and (**b**) z-axis versus time under 1 Hz sinusoidal current input.

First, the temperature of the coils under 1 Hz sinusoidal current was measured to examine the feasibility of long-term use. The measurements were stopped after 15 min (i.e., the maximum desired continued working time). In addition, the temperature was measured at middle parts of the coils. Figure 6 shows the time histories of temperatures for the coils. Results indicate that under an input current of 4 A, the temperatures of the coils along the x-axis (or y-axis) and z-axis were raised continuously, which were measured as 92.4${}^{{}^{\circ}}C$ and 78.7 ${}^{{}^{\circ}}C$ at 15 min, respectively. Therefore, the developed alternating electromagnetic field generation system can be used in the long term when the input current is below 4 A under 1 Hz sinusoidal signal.

### 4.3. Performance Test of the Field Generation

**Figure 7:**
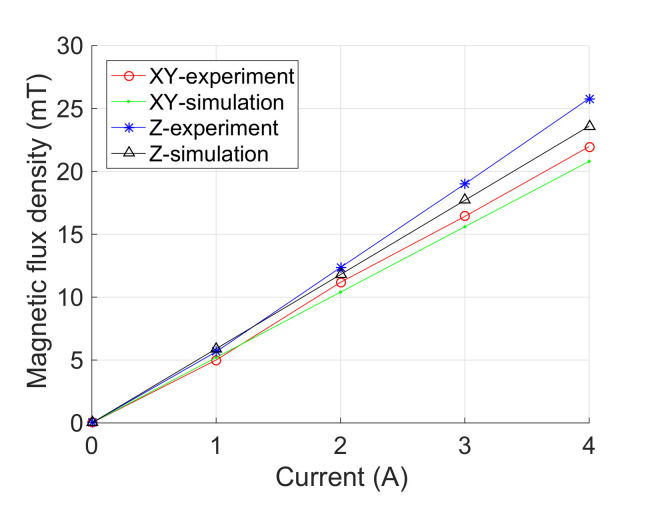
Experimental results of the magnetic flux densities at the center point versus input current.

Second, experimental tests were conducted to evaluate the magnetic-field-generation performance of the developed prototype. Figure 7 indicates that the magnetic flux density of the coils along each axis is in proportion to its input current, which is consistent with the prediction of theoretical model. The maximum magnetic flux densities of the coils along the x-axis (or y-axis) and z-axis were measured as 22.0 mT (i.e., mean value for the coils along the x-axis and y-axis) and 25.8 mT at the center point, respectively. The experimental results of $B_{max-xy}$ and $B_{max-z}$ are 9.3% and 5.8% larger than the simulation results, respectively.

Under an input current of 4 A, the maximum magnetic flux densities generated at the reference point were also measured. $A_{ang-xy}$ and $A_{ang-z}$ were derived as 0.990 and 0.990, which are 0.7% and 0.8% lower than the simulation results, respectively. Thus, the inclined angle between the direction of magnetic field at the center point and reference point is less than $8.1^{{}^{\circ}}$. Moreover, $A_{grad-xy}$ and $A_{grad-z}$ were obtained as 0.50 T/m and 0.52 T/m, which are 0.33 T/m and 0.04 T/m larger than the simulation results, respectively. The discrepancy is mainly induced by the manufacturing error, assembly error, and measurement error in the experiments.

**Figure 8:**
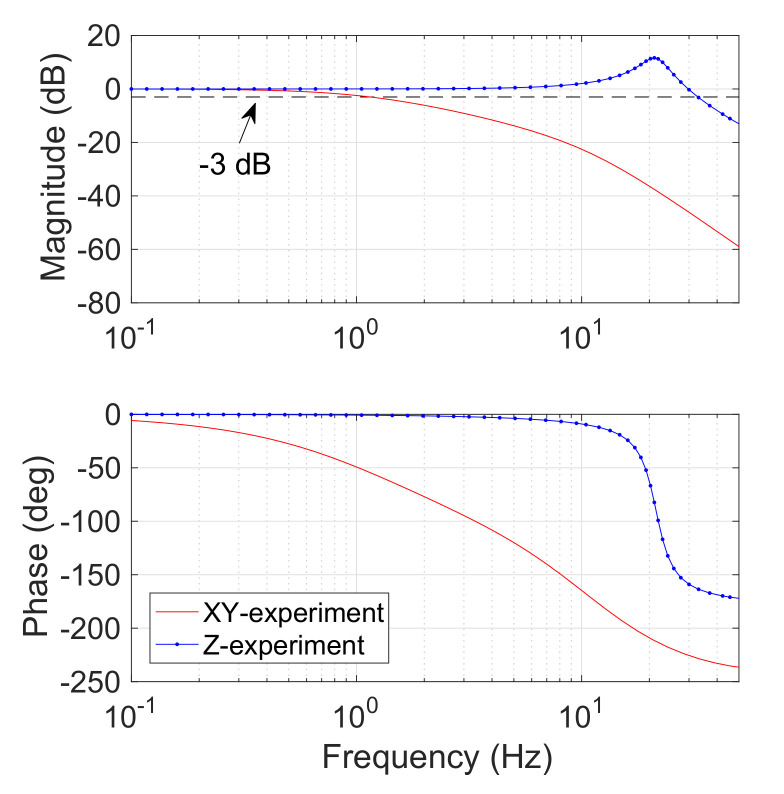
Bode diagram of the proposed alternating electromagnetic field generation system from system identification.

To evaluate the system performance of frequency response, transfer function model of the developed system was identified based on experimental results of step response with 4 A current input (with 50 Hz sampling frequency). According to the results of system identification, a Bode plot was obtained as shown in Figure 8. With open-loop control, the cut-off frequencies of the coils along the x-axis (or y-axis) and z-axis were derived as 1.2 Hz and 32.9 Hz, respectively. It is found that the cut-off frequency of the coil along the z-axis is much larger than those of the coils along the x-axis (or y-axis). This is caused by a relatively large difference in their inductances. The results indicate that the performance of the fabricated prototype satisfies the requirements for the targeted application.

**Figure 9:**
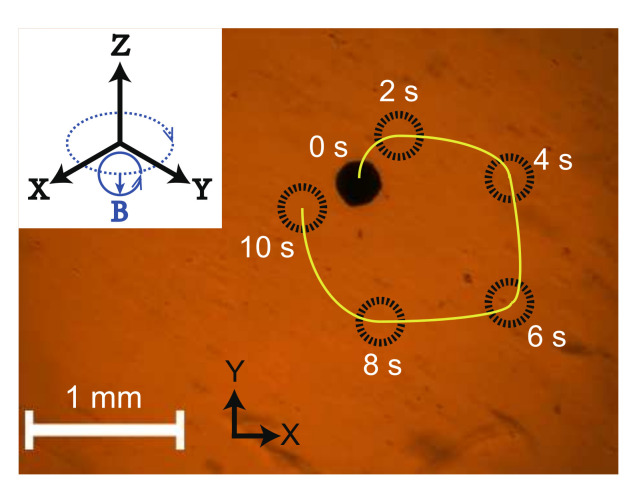
Snapshots of driving a magnetic micro-ball in a Petri dish filled with pure water. Black circle—position of the magnetic microball; yellow curve—actual trajectory; top-left inset—actuation magnetic field.

For demonstration, a magnetic microball with a diameter of 320 μm was driven to verify the effectiveness of the developed magnetic actuation system. The magnetic microball was fabricated by coating a layer of magnetic nanoparticles (NdFeB) on the surface of a gel spheroid. The magnetic microball was actuated for navigation by surface rolling in a petri dish filled with pure water. The servo amplifiers operate in current control mode for mitigating the hysteresis effect caused by the inductances. In this test, the rolling direction of the magnetic microball was varied over time, and the actuation magnetic field is shown in the top-left inset in Figure 9. The actuation magnetic flux density, rolling frequency, and direction variation frequency were assigned as 10 mT, 10 Hz, and 0.1 Hz, respectively. Figure 9 illustrates the snapshots (extracted from the Supplementary video file) of the magnetic microball in navigation process. The results indicate that the proposed system is effective for actuating the magnetic object to navigate in any direction by rolling motion. In addition, the magnetic microball did not move along a circle exactly, because the microball is not a perfect spheroid.

### 4.4. Discussion on the System Performance

To facilitate a comparison study, Table 3 tabulates the dominant performances of the proposed system and existing works. To assess the ability of the magnetic actuation system for generating a large magnetic field under the constraints of a certain hollow space and electrical power consumption, a figure of merit (FOM) is introduced for comparison. In particular, the FOM is assigned as the maximum magnetic flux density at the center ($B_{c}$) multiplied by the volume of hollow space ($V_{h}$), and then divided by the maximum total input power ($P_{\max-total}$) of the system as follows:

(17) $$\mathrm{FOM}=\frac{B_{c}V_{h}}{P_{\max-total}}\;\cdot \;\frac{1\;W}{1\;\mathrm{mT}\;\cdot \;\mathrm{mm}{}^{3}}$$

where $P_{\max-total}$ is determined as the sum of maximum instantaneous power for each coil. Table 3 indicates that the proposed system has a satisfactory FOM, even though the optimization was conducted under the constraints given in Table 1.

In the future work, the fabricated prototype system will be adopted for driving magnetic microrobots under the observation via an inverted microscope for biological application.

## 5. Conclusions

This paper presents the design, optimization, development, and testing of a new alternating electromagnetic field generation system for use with an inverted microscope. An optimization scheme is implemented by taking into account both both electric and geometry constraints. To obtain a more sophisticated and accurate optimization process, analytical models were established to determine the main parameters. According to the requirement of further application, MOGA method was conducted to optimally tune the system parameters. A prototype with the optimal parameters was fabricated for experimental tests. The results indicate that the parameter estimation is accurate, the optimal design is effective, and the performances of the developed system satisfy the requirements for targeted application. For demonstration, a magnetic microball was driven by the developed magnetic actuation system for navigation by rolling in a petri dish filled with pure water. In the future, the proposed system will be employed to actuate more untethered magnetic microrobots under the inverted microscope for biological studies.

## Figures and Tables

**Table 1:** Optimization objectives and constraints.

Input Parameters	
Constraint	$l_{c-xy}+g_{c-xy}\leq$ 140 mm
Constraint	$g_{c-xy}\geq g_{i-xy}$
Constraint	$2w_{c-xy}+d_{i-xy}\leq$ 40 mm
Constraint	$2g_{i-xy}\geq d_{i-xy}$
Constraint	$2g_{c-xy}\geq d_{i-xy}+2w_{c-xy}$
Constraint	$2g_{c-xy}\geq d_{a-z}+2w_{c-z}+$ 10 mm
Constraint	Input parameters $\subseteq R^{m}$
**Output Parameters**	
Objective	Minimize $L_{xy}$, high importance
Objective	Minimize $L_{z}$, high importance
Objective	Maximize min($B_{max-xy}$, $B_{max-z}$), medium importance
Objective	Minimize $A_{grad-xy}$, low importance
Objective	Minimize $A_{grad-z}$, low importance
Objective	Maximize $A_{ang-xy}$, low importance
Objective	Maximize $A_{ang-z}$, low importance
Constraint	min($B_{max-xy}$, $B_{max-z}$) ≥ 20 mT
Constraint	$A_{ang-xy}\geq$ 0.99
Constraint	$A_{ang-z}\geq$ 0.99

**Table 2:** Optimization results.

Parameter	Value	Parameter	Value
Input parameters			
$N_{lc-xy}$	120	$N_{wc-xy}$	8
$d_{i-xy}$	15 mm	$g_{c-xy}$	45 mm
$g_{i-xy}$	35 mm	$d_{c-xy}$	0.6 mm
$N_{lc-z}$	70	$N_{wc-z}$	5
$d_{a-z}$	44 mm	$d_{c-z}$	0.6 mm
Output parameters			
$B_{max-xy}$	20.8 mT	$B_{o-xy}$	5.2 mT
$I_{max-xy}$	4.0 A	$R_{xy-0}$	3.6 Ω
$A_{grad-xy}$	0.17 T/m	$A_{ang-xy}$	0.997
$L_{xy}$	132.6 mH		
$B_{max-z}$	23.6 mT	$B_{o-z}$	5.9 mT
$I_{max-z}$	4.0 A	$R_{z-0}$	3.1 Ω
$A_{grad-z}$	0.48 T/m	$A_{ang-z}$	0.998
$L_{z}$	5.5 mH		

**Table 3:** Performance comparison with existing alternating magnetic actuation systems.

Performance	This Work	[33]	[34]	[28]	[35]
Hollow space	7.54 × 10${}^{4}$ mm${}^{3}$	1.73 × 10${}^{6}$ mm${}^{3}$	3.04 × 10${}^{6}$ mm${}^{3}$	4.09 × 10${}^{3}$ mm${}^{3}$	-
Maximum magnetic flux density at the center point	22 mT	37.4 mT	14 mT	20 mT	6.5 mT
Maximum input current	4.0 A	15 A	9.2 A	20 A	3 A
Resistance of one coil	3.9 Ω (x,y);	6.7 Ω (x);	23.7 Ω (x);	1.3 Ω	10 Ω
	3.2 Ω (z)	9.3 Ω (y);	15.1 Ω (y);		
		5.4 Ω (z)	32.2 Ω (z)		
Number of electromagnetic coil ($N_{e}$)	5	6	6	8	4
Maximum total input power	300.8 W	9630 W	12,018.9 W	4160 W	360 W
Figure of merit (FOM)	5514.6	6718.8	3541.1	157.34	-

## Data Availability

Not applicable.

## Supplementary Materials

The following supporting information can be downloaded at: https://www.mdpi.com/article/10.3390/mi13040542/s1, Video S1: Experimental result of driving a magnetic micro-ball in a Petri dish filled with pure water.
